# Structural Basis for Epitopes in the gp120 Cluster A Region that Invokes Potent Effector Cell Activity

**DOI:** 10.3390/v11010069

**Published:** 2019-01-16

**Authors:** William D. Tolbert, Rebekah T. Sherburn, Verna Van, Marzena Pazgier

**Affiliations:** 1 Infectious Diseases Division, Department of Medicine of Uniformed Services University of the Health Sciences, Bethesda, MD 20814, USA; william.tolbert.ctr@usuhs.edu (W.D.T.); rebekah.sherburn.ctr@usuhs.edu (R.T.S.); 2 Department of Biochemistry and Molecular Biology of University of Maryland School of Medicine, Baltimore, MD 21201, USA; vvan@umaryland.edu

**Keywords:** HIV, structure, ADCC, vaccine, A32, C11

## Abstract

While a number of therapeutic options to control the progression of human immunodeficiency virus (HIV-1) now exist, a broadly effective preventive vaccine is still not available. Through detailed structural analysis of antibodies able to induce potent effector cell activity, a number of Env epitopes have been identified which have the potential to be considered vaccine candidates. These antibodies mainly target the gp120 Cluster A region which is only exposed upon viral binding to the target cell with epitopes becoming available for antibody binding during viral entry and fusion and, therefore, after the effective window for neutralizing antibody activity. This review will discuss recent advances in the structural characterization of these important targets with a special focus on epitopes that are involved in Fc-mediated effector function without direct viral neutralizing activities.

## 1. Introduction

According to the World Health Organization, HIV-1 has infected more than 70 million people, resulting in over 35 million deaths, including 1 million in the last year alone [1]. The virus achieves this, in part, by circumventing the immune system, which it then attacks and debilitates over time. Despite this, the immune system is able to clear infected cells and hold the virus at bay, often for years, before the onset of AIDS. One of the main targets for the immune system is the only viral protein on the surface of virions: the HIV Envelope trimer (Env), a heterotrimeric assembly of three copies of an internal gp41 and an external gp120 glycoprotein [2]. Antibodies can block the virus by recognizing and binding to epitopes within HIV Env, followed by utilization of either of two main mechanisms: inactivating the virus directly via neutralization or indirectly though Fc-mediated effector functions. Both methods are dependent upon the antibody placement on Env and the time of action during HIV’s life cycle. In general, neutralization requires binding of antibody early enough to prevent viral interactions with the target cell receptor CD4, with co-receptors CCR5 or CXCR4, and/or to lock Env’s conformation to prevent target cell fusion [3]. Fc-mediated effector functions are less dependent on when an antibody binds during HIV’s life cycle and more dependent on binding to sites that permit effector cell access to the Fc portion of the antibody [4]. 

Env employs a number of strategies to avoid immune system recognition including heavy glycosylation, high sequence variability, and conformational masking [5,6,7,8,9]. On the mature virion, only a small number of Env trimers exist [10], of which only some assume the ground, fusion competent state - referred to as the prefusion, closed conformation [11]. Antibodies targeting this Env conformation are typically neutralizing but are limited by the few binding opportunities to the target which is tightly shielded by glycans and undergoes subtle but constant structural rearrangements to transiently expose decoy epitopes. To bypass these obstacles, neutralizing antibodies have evolved several unusual structural features that allow them to precisely bind the moving target of HIV Env. It is clear that most of the effective neutralizers require at least one long complementary determining region (CDR), most often the inherently longer heavy chain CDR H3 (CDR H3) [12,13] and/or post-translational modification of CDRs to permit effective Env recognition [14]. Antibodies with long CDR H3s represent only a small fraction of the naïve antibody repertoire and require several rounds of selection and somatic hypermutation (SHM) to reach the lengths and high level of mutation in the heavy chain variable domain (often above 30%) required to function effectively against HIV-1 [15,16]. Effective neutralizers, therefore, only develop in chronically-infected individuals who have been exposed to HIV over a long period of time and even then, only in around 50% of chronically infected individuals [17]. 

Another obstacle in mounting an effective neutralizing humoral response is related to the error prone replication of HIV [18]. The amino acid sequences for the few Env regions that constitute neutralization targets are constantly changing during the course of infection and differ significantly between HIV strains, making antibodies to these regions only effective against a small subset of strains [19]. The exceptions to this are regions required for Env stability or function whose mutation impedes or prevents viral transmission with the best example of this being the CD4 binding site [20,21]. Antibodies binding the CD4 binding site can be effective and broad HIV neutralizers but are difficult to elicit by the immune system. They exhibit several structural features which result only from long-term affinity maturation processes such as rare insertions and deletions, to avoid steric clashes, and a long CDR H3 to reach deeply inside the CD4 binding site, which is roughly half the width of the antigen binding arm [22,23]. Strategies to mimic this long-term processing of CD4 binding site-specific antibodies are still a challenging task in HIV vaccine development [24,25]. 

According to basic definition, neutralization is the loss of virus infectivity caused by antibody binding and therefore neutralizing antibodies must recognize and bind at the Env epitopes/sites that are directly engaged in the functional mechanism of viral entry and spread [26]. However, in humoral responses to HIV, a large number of Env antibodies are generated to epitopes that only become accessible for antibody recognition in the later stages of viral entry or when the fusion process has already begun. These antibodies are therefore unable to impact the virus through a direct neutralization mechanism but rather constitute important components of the humoral response that acts based on Fc mediated effector functions such as antibody-dependent cellular cytotoxicity (ADCC). In ADCC, an antibody plays the role of a bridge with the antigen binding fragments (Fabs) engaging the HIV bound or infected cell and the crystallizable fragment (Fc) available for binding by effector cell Fc gamma receptors (FcγR) to mediates cytotoxic killing [27]. In theory, antibody binding to any exposed region of Env on the target cell can potentially function as an anchor for FcγR binding and effector cell activation, but certain regions are better targets for ADCC mechanisms than others. In the ADCC process, regions of Env with high conservation that permit easy access for effector cells are predicted to be the best targets for the effective control of HIV-1 [28].

Recent and accumulating evidence points toward conformational epitopes within the constant region 1 and 2 (C1-C2) of the gp120 domain as being major Env targets for antibodies capable of potent ADCC without direct neutralization activity, as measured using TZM-bl assays [29,30,31]. They are collectively referred to as Cluster A region epitopes [32,33] with the monoclonal antibodies (mAb) A32 and C11 being canonical examples that recognize non-overlapping epitope structures within this region [34,35,36]. The Cluster A epitopes become exposed upon the binding of the Env trimers to the target cell CD4 [37] and persist on freshly infected cell surfaces for extended periods of time post-infection [32,33]. These epitopes are also detected on the surface of HIV infected cells which retain detectable levels of CD4, required to trigger the Env trimers on the budding cell [38,39] and in the process of cell to cell spread. In the prefusion, closed Env trimer the Cluster A region of gp120 is buried at the gp41-gp120 interface directly contributing to the trimer assembly [35,40]. As part of the trimer interior these sites are not protected by the glycan shield and show a high degree of structurally enforced sequence conservation and, therefore, constitute highly vulnerable Env sites for humoral responses when exposed. Prolonged exposure of these sites on the surfaces of target cells during viral entry and on infected cells post-CD4 binding render them highly immunogenic. Antibodies specific for these targets are frequently elicited during HIV-1 infection and ADCC to the Cluster A region has been shown to be the major ADCC response in chronically infected individuals [31,32,39]. The presence of these antibodies also correlates with reduced infant infection risk and reduced mortality in infected infants in passive transfer from mother to child [41,42]. Interestingly, a number of antibodies against the Cluster A A32 epitope sub-region were isolated from vaccinated subjects in the RV144 vaccine trial, in which ADCC activity was correlated with protection in subjects with low IgA responses to gp120 [43]. The implications of the A32-like antibody response in the ADCC protective effect in the RV144 trial has renewed interest in these targets and stimulated research aimed at understanding their molecular basis. 

**Figure 1:**
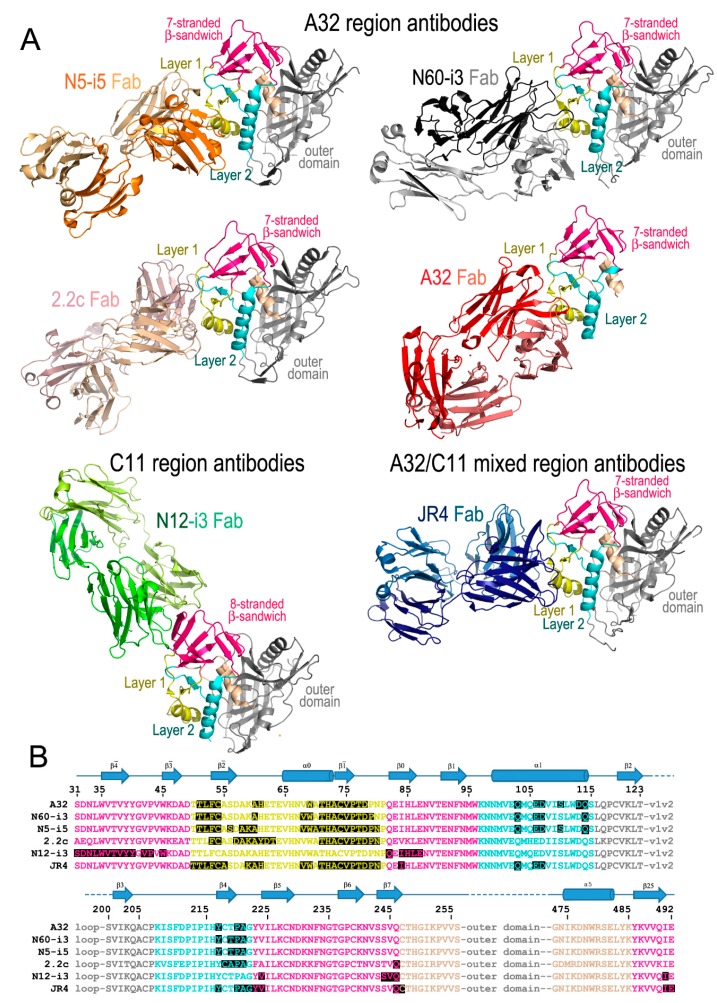
Cluster A antibodies in complex with gp120 antigens. (**A**) Structures shown are of: N5-i5Fab-gp120_93TH057_core_e_-d1d2CD4 (PDB code: 4H8W), N60-i3Fab-gp120_93TH057_core_e_-M48U1 (4RFO), 2.2cFab-gp120_YU2_core_e_-M48U1 (4R4F), A32Fab-gp120_93TH057-_ID2 (4YC2), N12-i3Fab-gp120_93TH057_core_e_+N/C-M48U1 (5W4L), and JR4Fab-gp120_93TH057_core_e_-M48 (4RFN). For each complex only the gp120 and Fab molecules are shown as ribbon diagrams. Images were generated with Pymol (The PyMOL Molecular Graphics System, Version 2.0 Schrödinger, LLC, San Carlos, CA, USA). (**B**) The binding footprint of each antibody highlighted in black. Colored text corresponds to the areas depicted in A; pink: seven-/eight-stranded β-sandwich, yellow: gp120 mobile layer 1, light blue: mobile layer 2, beige: layer 3, and grey: the gp120 outer domain. gp120 secondary structure elements are depicted above the sequence as arrows for β-strands and cylinders for α-helices.

At least three distinct epitope targets can be assigned within the Cluster A region (32,33) by cross-competition with the mAbs A32 and C11 [44]. The A32 and C11 epitope regions are defined as Env sites within the Cluster A region that are recognized by antibodies which completely abrogate binding of A32 and C11 to CD4-triggered gp120, respectively. The third epitope region is defined as the A32-C11 mixed region and is recognized by antibodies competing with both A32 and C11 for binding to the Env antigen. Below, the detailed characteristics of these important epitope targets on Env are presented with the specificities for the A32, C11 and A32-C11 mixed epitope regions in relation to each other and the Env conformation to which they bind (Figure 1).

## 2. The A32 Epitope Region

The core of the HIV-1 gp120 glycoprotein is comprised of three major structural domains: the outer domain, the inner domain, and the bridging sheet. The outer domain contains the CD4 binding site and is exposed in the prefusion, closed HIV trimer while the inner domain is occluded and packs against gp41 inside the trimer [45]. The inner domain is organized into three sub-domains, referred to as layers, that mediate binding to gp41 and undergo conformational changes during the process of trimer triggering by CD4 [35,40]. Layers 1–3 of the inner domain are connected through a structurally rigid platform of the seven-stranded β-sandwich that, together with β-strands from both the N and C-termini of gp120, cap the inner domain from the top [35,40]. Studies utilizing mutagenesis and antibody cross-competition with mAb A32 initially mapped the A32 epitope region to the gp41 interactive face of gp120, most likely within the topological layers of the gp120 inner domain in the C1 region [36,46]. Binding of A32 to the CD4-triggered gp120 was also shown to be affected by mutations in all three layers of the inner domain, the seven-stranded β-sandwich, the outer domain, and the β20-β21 loop of gp120 [39,40,46].

These initial mappings enabled basic epitope assignments, the precise and exact location of the A32 epitope region could only be defined once the structures of complexes formed between the A32-like antibodies and an appropriate Env antigen became available. Currently, these include crystal structures of complexes formed between CD4-triggered gp120 cores and antigen binding fragments (Fabs) of four antibodies specific for the A32 region: three A32-like antibodies (N5-i5, N60-i3, and 2.2c) and the prototype antibody A32 itself (Figure 1). mAbs N5-i5 and N60-i3 were isolated from a population of elite controllers termed natural virus suppressors (NVS) [47,48,49,50] and 2.2c was derived by an Epstein–Barr virus (EBV) transformation of peripheral blood B cells from an HIV-1-infected subject (RW/92/13) [51]. Figure 1A,B shows the A32 epitope region, as defined by these structural analyses, shown together with detailed epitope footprints of A32, N5-i5, N60-i3 and 2.2c. The A32 region maps exclusively within the inner domain of gp120 and includes residues within the inner domain mobile layer 1 (the β$\bar{2}$ and β$\bar{1}$ strands, α0 helix, and β$\bar{2}$-α0 and β$\bar{1}$-β0 connecting coils; residues 51–54, 56–61, and 68–80) of the C1 region and layer 2 (the α1 helix, β4 strand, and β4-β5 connecting coil; residues 103, 106–107, 110, 113–114, 217, and 219–221) of the C2 region. The residues of the gp120 outer domain and the N-, C-termini of gp120 were not found to contribute to A32 region epitopes. 

The gp120 surface which contributes the A32 epitope region in the CD4-triggered Env is relatively flat, glycan-free, and moderately electronegative [32]. Structural and sequence analysis revealed that common characteristics of the Fab region of antibodies targeting these epitopes have a moderate length of CDR H3 loop (10–13 residue long) and a positive average net charge of combined CDR H1, H2 and H3-s [32]. In all cases, the antibody-Env antigen interface is stabilized by a network of electrostatic interactions that play a pivotal role in the mechanism by which A32 region antibodies interact with their cognate epitopes. Additionally, for each antibody, except N5-i5, the CDR H3 is central to binding as it contributes most of the antibody contacts. N5-i5 instead attaches mainly through its relatively long CDR H2 (17-aa long). Although the majority of contacts to A32 region antibodies are provided through CDR H3, no significant correlation has been described between CDR H3 length or degree of V_H_ affinity maturation and ADCC activity for antibodies targeting the A32 region epitopes [52]. Conversely, with antibodies involved in neutralization, effective potency is often associated with unique or unusual structural features such as very long CDR H3 and a high degree of affinity maturation [53,54,55,56,57,58]. This, therefore, highlights important functional differences between antibodies to the A32 region and neutralizing antibodies. 

## 3. The C11 Epitope Region

Similar to the A32 region, the C11 epitope region was initially mapped by antibody cross-competition and mutagenesis analyses. These studies placed the C11 epitopes at the gp41 interactive region of gp120 within the C1, C5, and the seven-stranded-β-sandwich (epitopes described in [34,35,36]). More recently, the first structure of a C11-like antibody, N12-i3, in complex with gp120 has been determined [59], enabling a detailed mapping of this epitope region within the context of the CD4-triggered gp120 antigen (Figure 1A,B). As predicted, N12-i3 bound to the seven-stranded β-sandwich region of the gp120 inner domain but also contacted residues of the gp120 N-terminus in a new, previously unreported conformation. In the N12-i3-bound state, the N-terminus of gp120 docks onto the seven-stranded β-sandwich of the gp120 inner domain to form an eight-stranded β-sandwich. In the eight-stranded β-sandwich, residues 31–42 of the gp120 N-terminus form a new β$\bar{4}$ strand that packs parallel to the β0 strand of the seven-stranded β-sandwich [59]. N12-i3 binds to the edge of the newly formed eight-stranded β-sandwich, making several contacts with the N-terminus but also reaching above and below the β-sandwich to extend its footprint, relying mostly on hydrophobic interactions for stability. Residues 42–43, 45, and 84–87 from the gp120 C1 region, 224, and 244–246 from the C2 region, and 491 from the C5 region of the eight-stranded β-sandwich form the majority of the C11 binding contacts within the CD4-triggered gp120. While the prototype antibody C11 has a slightly longer CDR H3 (22-aa), N12-i3 has a shorter CDR H3 of 10-aa, comparable to the A32 region CDR H3 lengths. This unique conformation of gp120 in the N12-i3 bound state has not been described previously for any of the monomeric gp120 or Env trimers studied in either the unbound or Fab-triggered states and is most likely emblematic of the late stages of the HIV-1 entry process.

## 4. The A32-C11 Hybrid Epitope Region 

The only structure that has been determined to date of an antibody in complex with gp120 that displaces both A32 and C11 in competitive ELISAs is the macaque antibody JR4 [60]. The structure is strikingly similar to known A32-like antibodies with a few key differences; JR4 binds both mobile layers 1 and 2 (at residues 50–55, 59–61, 68–69, 71–80, and 82 in layer 1 and 103, 106–107, 217, and 220–222 in layer 2), utilizing many of the same gp120 residues as A32 and other A32-like antibodies, however, in contrast to those antibodies JR4 also contacts layer 2 with its heavy chain CDR H1 (Figure 1A,B). While seemingly a minor difference, this shifts the footprint to make more contacts within the seven-stranded β-sandwich region of the gp120 inner domain (residues 84, 223–224, and 246–247 and residue 492 of the C terminus). This shift is enough to interfere with binding of N12-i3, C11 and other C11-like antibodies in SPR and FCS experiments utilizing Fabs, confirming the initial competitive ELISA results using IgGs [60]. When antigen complex structures of JR4 and N12-i3 are compared, there are gp120 residues common to both epitopes (e.g., residues 84, 224, and 246–247) and others that are in close proximity (residue 492 from JR4 and 491 from N12-i3).

## 5. Cluster A Exposure during the CD4 Induced Env Trimer Opening

**Figure 2:**
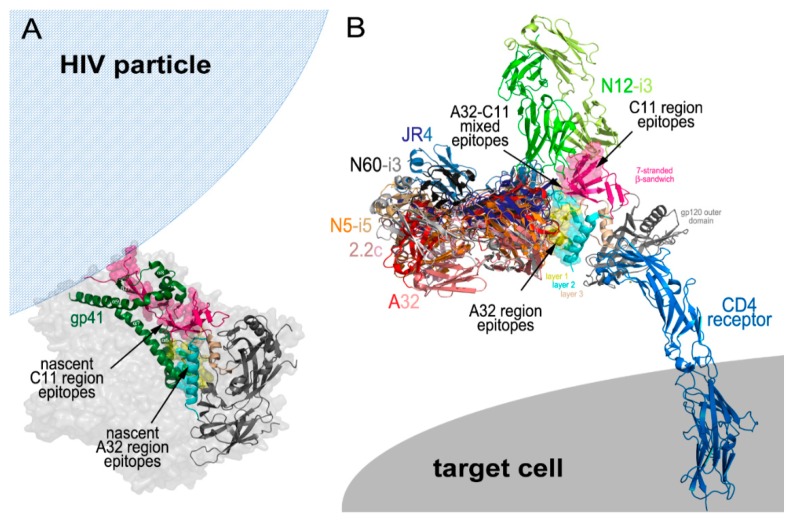
HIV-1 Epitopes. (**A**) Structure of a native, un-triggered virion-associated HIV-1 trimer showing the location of nascent C11 region and A32 region epitopes enclosed in the center of the non-CD4 bound trimer. (**B**) Structure of the HIV-1 trimer in the CD4 receptor bound confirmation. Binding exposes the C11, A32, and A32–C11 mixed epitope regions. The binding sites of all antibodies discussed in this review are depicted. Images were generated with Pymol (The PyMOL Molecular Graphics System, Version 2.0 Schrödinger, LLC, San Carlos, CA, USA).

The Cluster A epitopes become available for antibody recognition as Env transition state structures are exposed following engagement of the Env trimer with host receptors during viral entry [32,33] and on infected cell surfaces after cis [39] or trans [37] triggering by CD4 (Figure 2). The Cluster A region and the nascent non-overlapping sites for the A32 and C11 epitopes are buried within the untriggered, prefusion HIV trimer and consist of gp120 residues that contribute to the gp120-gp41 interface and stabilize the trimer by direct interactions with residues of the gp41 protomer. When analyzed in the context of CryoEM tomograms of a native, un-triggered virion-associated HIV-1 trimer and recently resolved crystallographic and Cryo-EM structures of a cleaved, soluble SOSIP gp140 trimer [5,6], the nascent A32 epitope region is located in the center of the prefusion trimer and interacts directly with the α7 helix and partially with the loop connecting the α6- α7 helices of gp41 [52] (Figure 2A). 

In contrast, the nascent C11 epitope region is located at the bottom of the prefusion trimer, proximal to the viral membrane and is directly involved in interactions with helices α6, α7, α8, and α9, and β-strand β27 of gp41. In addition, the N- terminus of gp120, which in the C11-bound state will form the 8th strand of the eight-stranded β-sandwich, is held by a triple-tryptophan clasp consisting of tryptophans 623, 628, and 623 of gp41 [7] (Figure 2A). As suggested in [61], the A32 and C11 region epitopes become available for antibody recognition sequentially during the process of the HIV trimer opening induced by cell surface CD4 attachment, therefore, are emblematic of two states in the series of conformational changes the Env trimer undergoes in the entry process (Figure 2B). Current evidence points toward the A32 epitope becoming available during a middle stage of viral entry and the C11 epitope at a late stage of viral entry. Unlike the co-receptor binding site recognized by canonical antibodies, such as 17b or 412d, the A32 epitope region is not exposed on virions by soluble CD4 [62,63]. These epitopes instead require the membrane bound form of CD4 for exposure, suggesting the requirement of additional energy to expose this part of the trimer interior [52]. Based on the location of the nascent A32 region within the un-triggered prefusion HIV trimer, A32 targets will be fully formed and accessible for antibody recognition only after a substantial displacement of gp120 protomers from gp41. This most likely will require a partial disassembly of the trimer to assume a conformation which has not yet been demonstrated by any structures of virion-associated or SOSIP HIV trimers bound with soluble CD4. In all known structures of this type [64,65,66,67] and also of trimers bound with both soluble CD4 and the co-receptor binding site antibody [45,68], the A32 region epitopes are partially buried at the trimer interface and not accessible for antibody recognition. This evidence suggests the possibility that the A32 epitope region is exposed only after the co-receptor binding site is fully formed and thus represents a middle stage of the entry process. 

In contrast, all evidence indicates that the C11 epitope region only becomes exposed during the final stages of viral entry, after the dissociation of gp41 from gp120 and is, thus, indicative of a late entry stage. The N- and C-termini of gp120 are integral elements in stabilizing the Env trimer in its closed, prefusion conformation and are engaged in a gp41 clasp [7]. Only upon detachment of the gp120-gp41 subunits and release of the gp120 N-terminus from the clasp can the eight-stranded β-sandwich and the C11 epitope region be fully formed and available for antibody recognition. Most likely at this stage, the gp41 cell fusion machinery has been activated and the gp41 collapse to a six-helix bundle begun with cell fusion imminent, if not complete. Exposure of the C11 epitope before or after cell fusion depends on how Env activation occurs; symmetrically or asymmetrically. In the former case, the C11 epitope only forms after cell fusion while in the latter there remains the possibility that the C11 epitope can be formed on one or more subunits before the cell fusion process has begun. In either case, the C11 epitope remains accessible on the cell surface until gp120 is released from the cell surface CD4 or the gp120-CD4 complex is internalized by the target cell. Synchronized cell fusion experiments suggest that Cluster A epitopes are exposed upon virion attachment and can remain exposed on the cell surface hours after initial exposure with the A32 epitope disappearing before the C11 epitope [33].

### Cluster A Region Epitopes as ADCC Targets

ADCC and other Fc-mediated effector cell functions have been implicated in protection from HIV infection as well as in the control of infection in a few elite HIV controllers [28,42,50,69,70,71,72,73]. The Cluster A region serves as a theoretically optimal target for a protective response and, therefore, has the potential for incorporation into a vaccine. The Cluster A targets are highly conserved and, unlike neutralizing antibodies, antibodies to this region do not require high levels of somatic mutation or unusual structural features that preclude their development. These epitope targets persist on freshly infected cell surfaces for extended periods of time post-infection [37] and promote potent ADCC in assays using virion sensitized cells [32]. Their persistence on cell surfaces in a model of cell-to-cell HIV-1 spread is unique and may explain, to some extent, why they are good targets for ADCC activities during the course of natural infection [74,75,76,77]. Since Cluster A epitopes are sequentially exposed during HIV entry, they are potentially also good targets for ADCC capable of inhibiting or blocking HIV-1 infection. These epitopes could also be important in the inactivation of HIV infected cells budding virus and contribute to the post-infection control of viremia by Fc-mediated effector mechanisms. However, the effectiveness of Cluster A directed ADCC in inactivating HIV infected cells is largely diminished by the HIV proteins Nef and Vpu [39]. These HIV-1 accessory proteins downregulate the cell surface levels of CD4 available to trigger the Env trimers emerging on the infected cell. It was shown that deletion of either Nef or Vpu increased infected cell recognition by 2–3 fold by mAb A32, but deletion of both increased recognition by 8-16 fold. This enhancement was abrogated by introducing a gp120 D368R CD4 binding site mutation, disrupting the recognition of infected cells by A32 and, therefore, ADCC [78].

Structural analysis has mapped the Cluster A region epitopes at the molecular level, defined the exact antibody contact sites on gp120 and described their three-dimensional shapes. Although most of the antibodies targeting these sites are uniformly able to mediate potent ADCC against targets exposed on trimeric Env after CD4 engagement, there is only one known example of an antibody which targets the A32 epitope region but is capable of only moderate ADCC. Comparative structural and functional studies of this antibody, known as 2.2c, to the potent ADCC mediator N5-i5, which targets an overlapping epitope structure in the A32 binding site, shed new light onto the role of fine epitope specificity and the mode of antibody attachment in the ADCC mechanism. In an RFADCC assay [52], 2.2c was capable of Fc-mediated effector function, but with noticeably lower potencies, as defined by two variables: % plateau cytotoxicity at saturating mAb concentrations and EC_50_ value using target cells (CEM-NKr-CCR5+) sensitized with a saturating dose of HIV-1Ba-L isolate gp120 in comparison to N5-i5, A32, and other A32-like antibodies. The analysis of the 2.2c Fab complex structure confirmed that 2.2c largely overlapped with N5-i5 in binding to the CD4-triggered gp120, but it recognized its epitope with some key differences. Specifically, 2.2c bound almost exclusively to mobile layer 1 of gp120 in the C1 region with no contacts to α1-helix and only a few contacts to the C2 region. In addition, the antibody approached the antigen from a slightly different angle and bound by swapping heavy and light chain relative to N5-i5. Binding analysis indicated that the mode of 2.2c attachment did not change the affinity of 2.2c for antigen, as compared to N5-i5, when measured by the SPR with antibody immobilized on sensor chip (affinity format). However, it did impair the ability of 2.2c to effectively bind to the antigen immobilized on the sensor chip (avidity format) and by inference, to recognize Env epitopes on the target cell. In addition, based on the inferred models of immune-complexes formed by 2.2c and N5-i5 at the target-effector cell interface, the Fc domain of 2.2c was orientated much closer to the putative target cell membrane than N5-i5, potentially impeding the effector cell access and the Fc-Fcγ receptor interaction [52]. 

The hypothesis of obstruction to effector function due to limited effector cell access to the Fc portion was confirmed in a set of experiments with the V_H_ and V_L_ domains swapped in variants of 2.2c and N5-i5 in which Fc domain orientations were interconverted. Surprisingly, the swapped variant of 2.2c which preserved the binding properties of wild type 2.2c but had the Fc orientation similar to that of N5-i5 showed an improved activity, but not to the levels seen for N5-i5 and other Cluster A antibodies. Conversely, the swapped variant of N5-i5 fully preserved the potent ADCC activities of the wild-type antibody. These findings strongly suggested there are two major determinates contributing to ADCC effectiveness to the A32 region epitopes; the ability of an antibody to form multivalent interactions with epitopes on the target cell surface (antibody-antigen crosslinking) and the Fc orientation in the immune complex. Although these observations were made for an antibody pair specific for the A32 epitope, these findings can be generalized and applied to other epitope targets indicating a dominant role for the precise epitope targeting and the mode of antibody attachment in ADCC responses. 

If orientation and accessibility of Fc (for interaction with effector cells) in the immune complex does contributes to the effectiveness of ADCC, as suggested above, the C11 epitopes of the Cluster A region should be optimal targets. As shown in Figure 2B, the C11 epitope region is located at the top of the gp120 molecule away from the target cell surface when in the CD4-bound state. Theoretically, C11 should be more accessible for antibody recognition than A32 and the mode of antibody attachment, as defined by heavy and light chain binding contacts, should not have a big effect on the formation of an effective target-effector cell complex. The N12-i3 binding mode orients its Fc domain away from the target cell membrane and it is likely that attachments to the C11-region through different binding modes will not have a big impact on accessibility of the Fc domain for an effector cell. In agreement with this, the activities of N12-i3, C11 and other C11-like antibodies are slightly higher in RFADCC assays as compared to other members of the Cluster A region [1]. While there is significant flexibility in CD4 and in the IgG hinge regions which correspond to uncertainty in the exact position of the Fc-FcγR in models based on the Cluster A epitope footprints, the C11 region IgG-Env antigen interaction likely represents an optimal geometry for effector cell access and activation.

## 6. The Role of ADCC in Vaccine Induced Protection

The recent vaccination strategy tested in the ALVAC-HIV/AIDSVAX-B/E RV144 vaccine trial, which showed an estimated vaccine efficacy of 31.2% in preventing HIV-1 acquisition, is of particular importance in the development of a HIV vaccine to induce protection in humans [79]. It suggested, for the first time, that vaccination can protect humans from HIV-1 infection, and that protection could be due to the generation of a blend of weakly neutralizing and non-neutralizing antibodies in the presence of modest CD4^+^ T cell responses [79,80]. The RV144 immunization regimen, comprising a canarypox ALVAC prime with an E.92TH023 gp120 membrane-anchored insert and an AIDSVAX B/E gp120 protein boost containing an 11-amino-acid (aa) deletion at the gp120 N terminus, induced a narrow array of antibodies specific for the V2 loop region [80], as well as antibodies specific for the CD4-inducible epitopes within the Cluster A region of gp120 [81]. While V2-specific mAbs were suggested to protect through both direct neutralization and Fc-mediated effector functions [82,83], the cluster A region specific mAbs were non-neutralizing and capable of potent ADCC [81]. Moreover, the cluster A specific antibodies synergized with V2 specific antibodies in ADCC responses, indicating crosstalk between these two epitope regions in delivering Fc-effector mediated responses [84]. Cluster A epitope specificity of antibodies in RV144 was assessed by measuring the levels of inhibition of plasma ADCC activity of vaccine recipients in the presence of the A32 Fab fragment [81]. Furthermore, most (19 of 23) of the ADCC-mediating mAbs isolated from vaccine recipients targeted multiple related but distinct conformational and A32-blockable epitopes [80,81]. These antibodies displayed low levels of V-heavy (V_H_) chain somatic mutation (0.5 to 1.5%) and mediated cross-clade ADCC activity against HIV-1 isolates of clades represented in the immunogen (Clade B and CRF01 AE) as well as Clade C, which was not represented in the vaccine [81]. Moreover, the ADCC responses to this region were greatly reduced by the presence of IgA mAbs incapable of NK cell-mediated effector function. These mAbs competed for the same Env binding sites, and therefore likely attenuated the protective vaccine efficacy due to a decrease in Fc receptor-dependent effector function [43]. The RV144 data hinted at an intriguing link between vaccine-mediated protection and ADCC, and provided the first strong evidence for the implication of the A32 region in vaccine induced protective ADCC responses in humans. 

With regard to studies in non-human primates (NHP), the evidence for a role of Cluster A epitopes in vaccine induced protection is even stronger. ADCC measured using the rapid fluorometric antibody-dependent cellular cytotoxicity assay (RFADCC) [85] and antibodies specific for the A32 and C11 epitopes of the Cluster A region, as well as for the co-receptor binding site, correlated with sterilizing heterologous protection against SHIV162p3 in NHPs [86] immunized with full length single chain (FLSC), a vaccine consisting of full-length gp120 stabilized in CD4-bound state [86,87,88]. Similar to the RV144 trial, no correlation between neutralizing activity and protection was observed in these studies, pointing to a role of Fc-mediated effector function to the Cluster A region in protection against SHIV-1 transmission.

## 7. Future Perspectives

It is clear that a pressing need for a preventative HIV vaccine exists and that we are closer than ever to overcoming the many obstacles required to create an effective therapy. The revelation that a degree of HIV protection could be achieved in the absence of broadly neutralizing antibodies in the RV144 vaccine trial spurred interest into the role of non-neutralizing antibodies and the epitopes to which they bind, which results in effector cell mediated killing of infected cells. Structural analysis of the gp120 cluster A region of the HIV envelope has greatly enhanced our understanding of the importance of antibodies generated to epitopes within this region. Through detailed analysis of isolated antibodies in complex with CD4 and gp120, the A32 region has been defined as a discontinuous site involving mobile layers 1 and 2 in the C1–C2 regions of gp120 in its CD4-bound state and C11 region as epitopes mapped exclusively within the eight-stranded β-sandwich of the inner domain of the C1-C2 regions of gp120 centered around the N-terminal eighth-strand. These two epitope regions are non-overlapping and are emblematic of two different steps in the process of CD4-induced opening of the trimer with C11 marking the late stages of trimer disassembly, most likely after complete gp120 detachment. This detailed map of the vital antibody binding epitopes will be invaluable for the development of future vaccine candidates for both the prevention and clearance of HIV.
